# Supplementary material for: Identification and functional analysis of non-coding regulatory small RNA FenSr3 in Bacillus amyloliquefaciens LPB-18
Source: PeerJ. 2023 May 15;11:e15236. doi: 10.7717/peerj.15236 (PMC10194069; doi:10.7717/peerj.15236)
Supplement: Supplemental Information 4 [file peerj-11-15236-s004.zip › KO/CK-vs-T1_map/map04013.html]

KEGG PATHWAY: MAPK signaling pathway - fly - Reference pathway


|  |  |
| --- | --- |
| **MAPK signaling pathway - fly - Reference pathway** |  |

[
Pathway menu
| Organism menu
| Pathway entry
| Show description
| User data mapping
]

The mitogen-activated protein kinase (MAPK) signaling cascades that are highly conserved from yeast to mammals use protein phosphorylation to convey signals intracellularly, and play a crucial role in mitosis, apoptosis, motility and metabolism. In fruit fly, three major classes of MAPK pathways have been distinguished with little or no redundancy, including extracellular signal-regulated kinase (ERK) / Rolled, c-Jun N-terminal kinase (JNK) / Bsk, and p38 kinase. Signals from growth factors or mitogens are dominantly transduced by the ERK pathway, whereas the JNK and p38 pathways are activated mostly by environmental stresses. The MAPK signaling cascades are typically linked to regulation of gene expression, because transcription factors are often the targets of regulatory phosphorylation events.

|  |  |  |
| --- | --- | --- |
| Reference pathway | 184% 150% 122% 100% 82% 67% 55% | 图片下载 |
